# Prioritizing Health in War and Conflict: The 2025 War in Iran and the Call for Global Peace

**DOI:** 10.34172/ijhpm.9428

**Published:** 2026-02-17

**Authors:** Azam Raoofi, Elham Ehsani-Chimeh, Amirhossein Takian

**Affiliations:** ^1^Center of Excellence for Global Health (CEGH), Department of Global Health, School of Public Health, Tehran University of Medical Sciences (TUMS), Tehran, Iran.; ^2^National Institute for Health Research (NIHR), Tehran University of Medical Sciences (TUMS), Tehran, Iran.; ^3^Department of Health Management, Policy, and Economic, School of Public Health, Tehran University of Medical Sciences (TUMS), Tehran, Iran.; ^4^Health Equity Research Center (HERC), Tehran University of Medical Sciences (TUMS), Tehran, Iran.

 The Israel-Iran war, intensified by the U.S. involvement, highlights the devastating toll of war on health systems, social unity, and public well-being. The conflict claimed 1062 lives and left 5800 injured, including 126 women, 47 children, 26 healthcare personnel and 28 emergency vehicles and facilities, ie, ambulances, hospitals, and healthcare centers damaged (Table).^1^ This paper reports the amplifying effects of pre-existing sanctions on the war harms, and applies the World Health Organization (WHO) Global Health and Peace Initiative (GHPI) as the guiding framework for the tailored policy recommendations.

**Table:** Statistics of Harms in Iran as of July 16, 2025^a^

**Category**	**Counts, No.**
Total casualties	6862
Total death	1062
Total injured	5800
Affected healthcare workforces	26
Killed healthcare workforces	6
Injured healthcare workforces	20
Killed women	126 (including 2 pregnant mothers)
Killed individuals under 18 years old	47 (the youngest was 2 months old)
Unidentified killed	24
Damaged ambulances	11
Damaged hospitals	7
Damaged healthcare centers	4
Damaged emergency stations	6

^a^ Figures compiled from official announcements by the Ministry of Health and Medical Education and news reports from July 1-16, 2025. These figures require independent verification by World Health Organization (WHO)/United Nations (UN)/International Committee of the Red Cross (ICRC) monitoring mechanisms.^2-4^

 Reported strikes on healthcare facilities, eg, on a hospital in Kermanshah, underscores the humanitarian toll and endangering civilians and healthcare workers.^5^ Such attacks violate Geneva Conventions and International Humanitarian Law, including WHO Resolution WHA65.20, and call for international accountability.^6^ Such destruction weakens health systems, jeopardizes population health, and compounds humanitarian crises by collapsing care, worsening mental health burdens, and forcing families into unsafe, crowded shelters.^1^ It is recommended that the WHO and the United Nations Human Rights Council initiate an independent fact-finding mission or monitoring mechanism to investigate health-targeted attacks and ensure accountability, where appropriate.^6,7^ Even before June 2025 hostilities, Iran’s healthcare system was strained by prolonged sanctions, intensified since 2018, which impeded access to essential medicines, diagnostics, and medical technologies, undermining care for millions and compromised the country’s ability to respond to public health emergencies, a situation worsened by COVID-19 and other crises.^8,9^ Sanctions have also exacerbated provincial health disparities, hindered chronic disease management, and weakened supply chains.^10^ The war further destabilizes this fragile infrastructure, elevating humanitarian and public health risks. Targeting healthcare facilities—seen in Syria and Gaza—exacts a heavy toll on vulnerable populations and undermines recovery and rebuilding.^11^ Sanctions weakened health system resilience during the June 2025 strikes by delaying medicines, degrading equipment, and reducing surge capacity—turning limited damage into excessive morbidity and mortality.

 Beyond immediate casualties, the conflict causes severe physical and mental harm. Survivors face limited access to care due to the destruction or repurposing of healthcare facilities, leading to long-term disabilities and worsening chronic illnesses, straining the health system.^1^ Persistent violence creates trauma, including chronic stress, anxiety, and post-traumatic stress disorder,^11,12^ undermining resilience as mental health services collapse amid facility closures and medicine shortages.^11^ In Gaza, since October 2023, and Syria, psychotropic drug stock-outs and facility shutdowns increased depression and post-traumatic stress disorder prevalence by up to 26.5%.^12^ In Iran, memories of the Iran–Iraq War (1980–1988) amplify collective trauma, shaping national responses.^13^ This may intensify current psychological distress, shape help-seeking behaviors, and constrain community resilience, thereby magnifying the mental-health burden following the recent strikes.^12,13^ With only 2.48 psychiatrists per 100 000 people in 2020^14^ and frequent disruptions, community-based mental health initiatives stall, deepening the care gap. The crisis also deepens the political fault lines, as the loss of military and security leaders adds strain to the social fabric and heightens national trauma. Recent aggressive actions raise concerns about long-term public health threats.

 Iran’s situation presents a distinct challenge compared to other contemporary health crises, eg, Ukraine and Gaza. Unlike Ukraine, which benefits from external aid, Iran, isolated by sanctions and political constraints, faces an uphill battle in accessing critical medical supplies and humanitarian aid.^9,10,15^ In Gaza, the blockade and conflict since October 7, 2023, have nearly collapsed the healthcare system.^16,17^ While both Gaza and Iran face systemic fragility under sustained shocks, Iran’s relative functionality potentially might be attributed to its decentralized structure and legacy of community health workers. Nonetheless, sanctions and conflict disrupt supply chains and threaten Iran’s care delivery. Iran’s health system has shown resilience in past emergencies, with nurses, physicians, and community health workers continuing to provide care during COVID-19 and sanctions.^9,10^ Yet sanctions plus conflict create a double vulnerability, intensifying supply-chain breakdowns and workforce pressures demanding targeted international intervention.

 Lessons from global crises emphasize that conflict’s toll goes beyond the battlefield. The World Health Summit 2024 panel on “Health in War and Conflict” urged integrated health governance to address injuries, infrastructure loss, and psychological distress. The WHO’s GHPI provides an operationally focused framework for this integration focusing on health as a bridge for peace and a platform for collaboration. To translate health into a practical unifying force three concrete mechanisms are focused on feasible measures derived from this approach are essential for maintaining essential health services under conflict and sanctions:

1. International Committee of the Red Cross (ICRC)/UN-negotiated health corridors to ensure uninterrupted supplies^18^; Building on the model used to deliver aid to besieged areas in Syria, this mechanism for Iran needs to be explicitly coupled with a dedicated financial channel, such as a UN-approved escrow account, to bypass sanction-related transaction barriers and ensure the direct flow of essential medicines and equipment to central medical warehouses.^9,11^
2. WHO Eastern Mediterranean Regional Office–coordinated cross-border hubs for logistics and resource mobilization^18^; The hub’s objective is to recover and maintain critical device functionality (ventilators, imaging, dialysis machines) and shorten repair times created by sanctions-era spare-parts delays. Feasibility rests on partnering with local biomedical engineering units and WHO’s regional logistics capacity. Short-term indicator: median time to repair critical biomedical equipment.^8,15^
3. Regular health diplomacy platforms to embed health in ceasefire and peacebuilding.^18^ The immediate, measurable outcome for Iran would be the negotiation of “health truces” for specific actions, such as facilitating the safe restocking of dialysis centers in strike-affected provinces or enabling mobile vaccination teams to reach border regions, directly addressing the critical gaps in chronic disease management and routine immunization exacerbated by the conflict.^13,18^

A paradigm shift must place health at the center of crisis response and long-term stability.^18^

## Moving Forward: Global Accountability and a New Peace Paradigm

 GHPI repositions health in peacebuilding initiatives and offers concrete applications to mitigate conflict’s health impacts (Figure). Despite decades of research, health in conflict zones remains compromised by global decisions. We urge world leaders to safeguard civilian health through stronger global health governance, recognizing that the costs of conflict—mortality, chronic illness, and mental health disorders—outweigh any short-term military gains. Studies link conflict to increased mortality, chronic illnesses, and mental health disorders.^11,19^ We advocate for health to be prioritized as a fundamental right during conflicts; protecting human rights and post-conflict resilience. To reduce casualties and rebuild trust, we invite the international community, policy-makers, and humanitarian agencies to act immediately on these tailored priorities (Figure).

**Figure:**
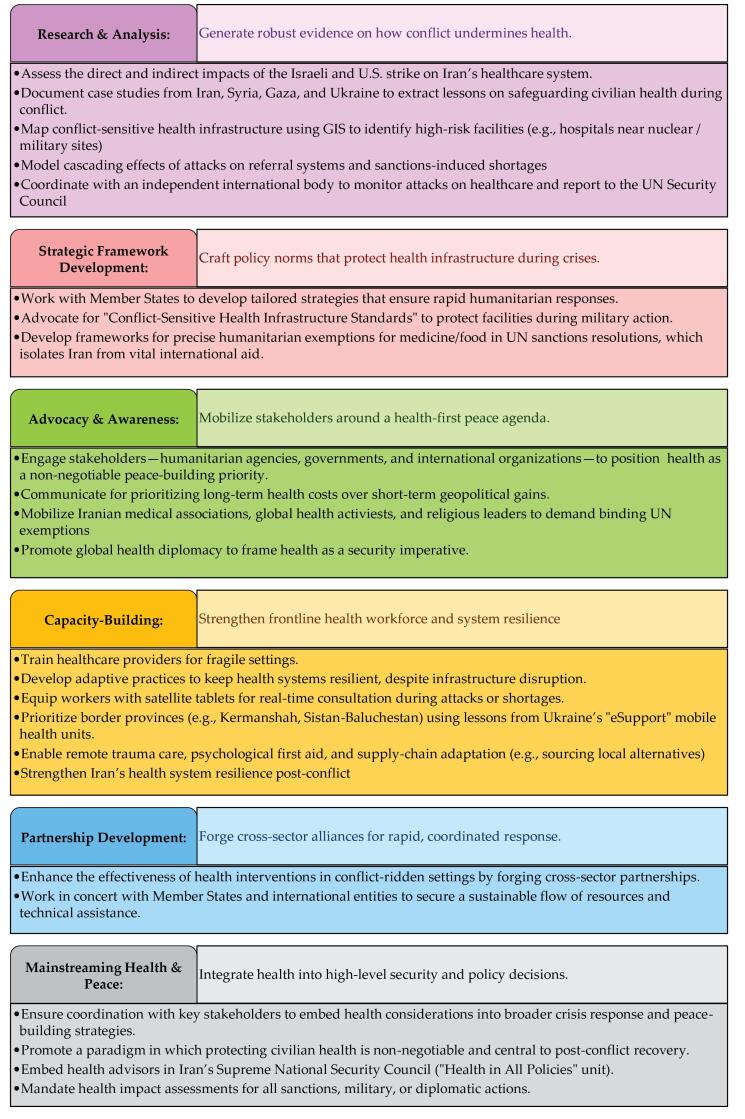
Global Health and Peace Initiative Tailored to Israel and U.S. Strike Against Iran (the figure is created by the authors). Abbreviations: GIS, geographic information system; UN, United Nations.

 Iran’s experience highlights the broader global health challenges posed by war. It reveals how marginalizing health and humanitarian concerns in politics yields lasting consequences. Ukraine and Gaza show neglect harms nations and health systems worldwide through globalization. Enforcing frameworks, enabling agile financing, and prioritizing health in sanctions can mitigate harm. The principles of the GHPI must become enforceable policy—health is a fundamental right that must be protected.

## Conclusion

 Health is a fundamental right that must not be subordinated to political agendas. The international community has the tools and obligation to protect health systems through three concrete actions. First, enforce health neutrality in conflict with robust multilateral mechanisms: the WHO, with the UN Security Council, should monitor attacks on medical facilities per WHA65.20, and the ICRC is expected to establish protected health zones around treatment centers. Second, sanctions must exempt essential medicines and equipment; the UN should reform policies to ensure uninterrupted humanitarian access, overseen by an independent body such as the Global Health Cluster. Emergency funds from the World Bank and WHO should bypass sanctions for health-infrastructure repairs. Third, mental health needs to be integrated into all humanitarian responses, scaling WHO programs with local health authorities to train workers in psychological first aid, especially in border regions; UNHCR and UNICEF must prioritize mental health services in displacement settings.

 Iran’s resilient healthcare system and its dedicated health workers demonstrates that health can unify communities if supported by targeted international action.

 In a world where nationalism often overwhelms global citizenship and human rights, we urge the international community to prioritize future generations over short-term gains. By incorporating a health-first perspective into conflict resolution and peacebuilding, we hope that tomorrow’s world can be built on pillars of human dignity and sustainable prosperity. Accountability for leaders is essential. To global health policy-makers, we emphasize: integrate conflict-sensitive health planning into emergency preparedness; ensure sanctions never obstruct humanitarian access to medicines and supplies; invest in scalable mental health infrastructure for war-affected populations; and uphold the neutrality of health systems in international law and diplomatic practice. Global governance must protect civilian health in practice.

## Disclosure of artificial intelligence (AI) use

 We used AI tools to enhance the readability and grammatical accuracy of this work. Specifically, we employed Microsoft Copilot with the prompt: “Please revise the text below grammatically,” and DeepSeek-V3 with the prompt: “Please shorten the text below,” to refine our initial draft. These tools were used for improving clarity, conciseness, and grammatical precision.

## Ethical issues

 Not applicable.

## Conflicts of interest

 Authors declare that they have no conflicts of interest.

## References

[1] Martini M, Valchi L, Massaro E, Parrella R, Orsini D (2024). War and health: the devastating impact of conflict on wellbeing and humanitarian crises. J Prev Med Hyg.

[2] Aftab News. [Announcement of Exact Figures of Martyrs and Wounded in the 12-Day War with Israel]. 2025. https://aftabnews.ir/004CfO. Accessed July 16, 2025.

[3] Shahraranews. The Latest Accurate Statistics of Martyrs and Wounded in the 12-Day War. 2025. https://shrr.ir/001Rjt. Accessed July 14, 2025.

[4] Ministry of Health and Medical Education. [Announcement No. 5 of the Crisis Information Headquarters of the Ministry of Health and Medical Education]. 2025. https://behdasht.gov.ir/ZhnPo.

[5] MacDonald A. Israeli Strike Damages Hospital in West Iran as Mutual Attacks Kill Dozens. Middle East Eye; 2025. https://www.middleeasteye.net/news/israeli-strike-damages-hospital-west-iran-mutual-attacks-kill-dozens. Accessed June 16, 2025.

[6] World Health Organization (WHO). WHO’s Response, and Role as the Health Cluster Lead, in Meeting the Growing Demands of Health in Humanitarian Emergencies. Geneva: WHO; 2012. https://apps.who.int/gb/ebwha/pdf_files/wha65/a65_r20-en.pdf.

[7] World Health Organization (WHO). Attacks on Health Care Initiative. WHO; 2020. https://www.who.int/news-room/questions-and-answers/item/attacks-on-health-care-initiative. Accessed September 21, 2025.

[8] Yazdi-Feyzabadi V, Zolfagharnasab A, Naghavi S, Behzadi A, Yousefi M, Bazyar M (2024). Direct and indirect effects of economic sanctions on health: a systematic narrative literature review. BMC Public Health.

[9] Takian A, Raoofi A, Kazempour-Ardebili S (2020). COVID-19 battle during the toughest sanctions against Iran. Lancet.

[10] Mohamadi E, Kraemer A, Majdzadeh R (2024). Impacts of economic sanctions on population health and health system: a study at national and sub-national levels from 2000 to 2020 in Iran. Global Health.

[11] David SD, Eriksson A (2025). Association between conflict intensity and health outcomes in contemporary conflicts, while accounting for the vulnerability and functioning of healthcare services. Confl Health.

[12] Hoppen TH, Priebe S, Vetter I, Morina N (2021). Global burden of post-traumatic stress disorder and major depression in countries affected by war between 1989 and 2019: a systematic review and meta-analysis. BMJ Glob Health.

[13] Behrouzan O (2018). Ruptures and their afterlife: a cultural critique of trauma. Middle East-Topics & Arguments.

[14] World Health Organization (WHO). Mental Health Atlas 2020 Country Profile: Iran (Islamic Republic of Iran). WHO; 2022. https://www.who.int/publications/m/item/mental-health-atlas-irn-2020-country-profile. Accessed August 14, 2025.

[15] D’Andrea SM, Fadul N, Dery M, Brim WL, Israel AM, Struminger BB (2024). Healthcare capacity strengthening in conflict settings through virtual emergency medical training and outreach: Ukraine and Sudan case studies. Front Public Health.

[16] Aljadba G, Elkhatib Z, Hamad K (2025). UNRWA’s health service in Gaza: challenges and response to the October 2023 crisis. East Mediterr Health J.

[17] Balkhy HH, Ghebreyesus TA (2025). A roadmap for healing Gaza’s battered health system. East Mediterr Health J.

[18] Abouzeid M, Elzalabany MK, Nuwayhid I, Jabbour S (2021). Conflict-related health research in Syria, 2011-2019: a scoping review for The Lancet - AUB Commission on Syria. Confl Health.

[19] Bürgin D, Anagnostopoulos D, Vitiello B, Sukale T, Schmid M, Fegert JM (2022). Impact of war and forced displacement on children’s mental health-multilevel, needs-oriented, and trauma-informed approaches. Eur Child Adolesc Psychiatry.

